# Ultrasonic Shear Wave Elasticity Imaging (SWEI) Sequencing and Data Processing Using a Verasonics Research Scanner

**DOI:** 10.1109/TUFFC.2016.2614944

**Published:** 2017-01

**Authors:** Yufeng Deng, Ned C. Rouze, Mark L. Palmeri, Kathryn R. Nightingale

## Abstract

Ultrasound elasticity imaging has been developed over the last decade to estimate tissue stiffness. Shear wave elasticity imaging (SWEI) quantifies tissue stiffness by measuring the speed of propagating shear waves following acoustic radiation force excitation. This work presents the sequencing and data processing protocols of SWEI using a Verasonics system. The selection of the sequence parameters in a Verasonics programming script is discussed in detail. The data processing pipeline to calculate group shear wave speed (SWS), including tissue motion estimation, data filtering, and SWS estimation is demonstrated. In addition, the procedures for calibration of beam position, scanner timing, and transducer face heating are provided to avoid SWS measurement bias and transducer damage.

## I. Introduction

Ultrasonic elasticity imaging has been under investigation since the late 1990's. It provides information about tissue stiffness, which is often associated with underlying pathological conditions [1]. An increase in tissue stiffness can be caused by the development of fibrotic tissue, as occurs in liver cirrhosis, or by an increase in tissue cellular density, as can occur with malignant tumors, inflammation, and other cancers. Many clinical applications of ultrasonic elasticity imaging have been reported to help clinicians diagnose and monitor diseases non-invasively based on stiffness contrast, particularly in breast and liver diseases [2], [3].

Many ultrasonic elasticity imaging techniques have been described in recent years including acoustic radiation force impulse (ARFI) imaging [4], shear wave elasticity imaging (SWEI) [5], supersonic shear imaging (SSI) [6], comb-push ultrasound shear elastography (CUSE) [7], and spatially modulated ultrasonic radiation force (SMURF) imaging [8]. These methods use acoustic radiation force from focused ultrasound beams to induce tissue motion and standard ultrasound imaging techniques to track the resulting tissue motion. ARFI imaging measures on-axis tissue displacement to determine relative differences in tissue stiffness, similar to the data generated with compressive strain imaging methods. The other methods quantify tissue stiffness by tracking tissue motion at locations offset from the ARFI excitation to determine the propagation speed of the associated shear wave. The conventional SWEI method uses multiple track beams to track the shear wave generated from a single ARFI excitation. The resulting shear wave speed (SWS) reflects the shear modulus between the track beams used in the estimation. SSI and CUSE methods use similar tracking configurations with different push focal configurations. These methods, which use multiple track locations for a given push location, are commonly referred to as multiple track location SWEI (MTL-SWEI) [9]. On the other hand, SMURF imaging uses multiple laterally-offset push beams and a single track beam. The SWS estimated in SMURF reflects the tissue properties between the push beams. This method is referred to as single track location SWEI (STL-SWEI) [9]. Measurements of SWS using STL-SWEI require multiple ARFI excitations, and thus deposit more energy into the tissue compared to MTL-SWEI measurements.

A measured SWS can be used to determine tissue properties assuming a mechanical model of the tissue. For a linear, elastic, isotropic, homogeneous and unbounded material, the SWS can be expressed in terms of the shear modulus *μ* and density *ρ* by the relation

(1) $$\text{SWS}=\sqrt{\frac{\mu }{\rho }}.$$

The density of soft tissue is typically assumed to be 1 g/cm^3^, and the SWS in units of m/s is equal to the square root of the shear modulus when it is expressed in units of kPa. In contrast, for a viscoelastic material, the shear modulus is a complex, frequency dependent quantity. Shear wave propagation in a viscoelastic material exhibits dispersion with a frequency dependent phase velocity and shear attenuation [10]. For an elastic material, the phase velocities are independent of frequency, and the measured group SWS is equal to the phase velocities. For a viscoelastic material, the measured group SWS represents an average of the phase velocities over the frequency content of the shear wave.

The Verasonics research system (Verasonics, Inc., Kirkland, WA) has been widely adopted by research labs to conduct ultrasound research. The Verasonics system is compatible with many Philips transducers, such as L7-4, P4-2, C5-2 arrays, and offers broad flexibility in sequence design. In addition, it provides direct access to the raw channel data from each element of the array, as well as a software beamformer to form ultrasound images. Many research groups have used the Verasonics system to design and implement novel elasticity imaging methods [11], [12], [13].

This manuscript describes the setup and parameter selection of ultrasonic MTL-SWEI sequences using a Verasonics system, and describes the data processing pipeline to calculate group SWS. Section II details the parameter space for a SWEI sequence and provides guidelines for defining these parameters in the Verasonics software. Section III presents procedures for calibrating beam positions and scanner timing to ensure accurate SWS measurements. Sections IV and V present suggested safety measurements for transducer protection and clinical studies. Section VI demonstrates the processing procedures to calculate group SWS, and Section VII discusses the recommended practices specifically for curvilinear arrays and other types of elasticity imaging methods. The list of variables and abbreviations that appear in this paper is summarized in Table I.

## II. Verasonics SWEI sequencing / Parameter specification

**Fig. 1:**
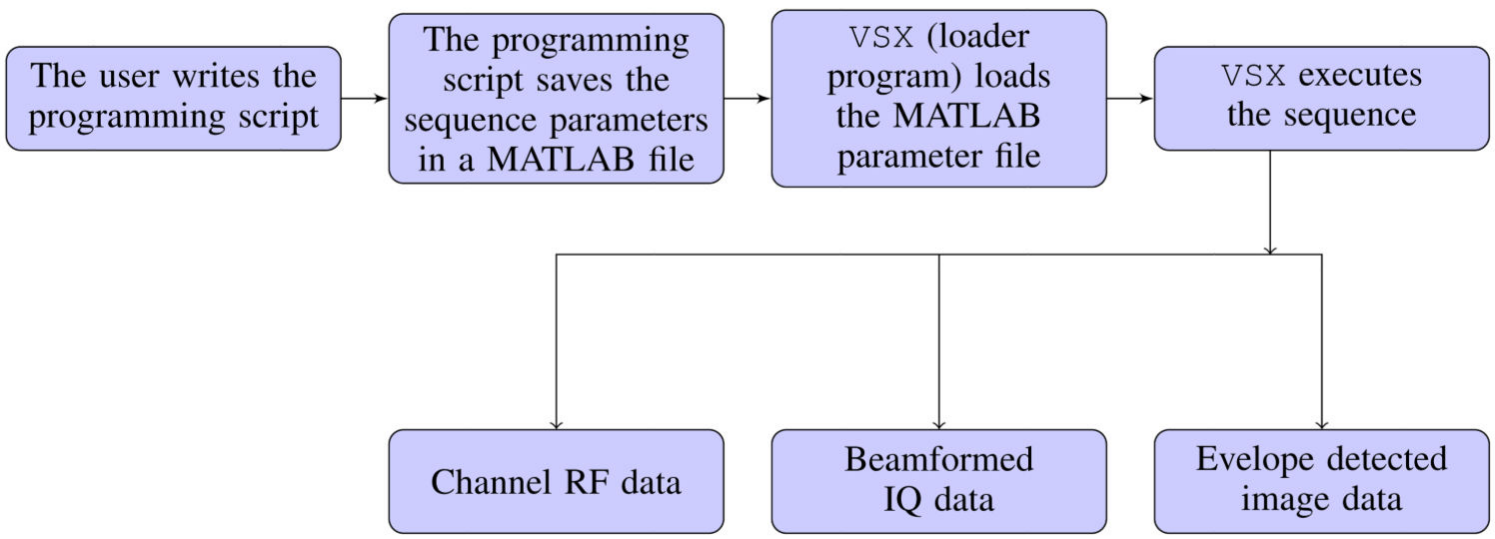
Verasonics sequence execution flow chart

Setup of the Verasonics system is performed in a MATLAB programming environment. To generate an imaging sequence, the user writes a programming script that generates a collection of objects that are loaded into the Verasonics scanner during runtime. The objects are defined using MATLAB structures. When the programming script executes, it defines the imaging parameters and the sequence of actions, populates them into a series of MATLAB structures, and saves all the information in the format of a MATLAB file. This MATLAB file can then be loaded into the system by a loader program (named vsx) to execute the sequence during runtime. Raw channel radio frequency (RF) data can be accessed after the completion of the imaging sequence. If the Verasonics beamforming is utilized, beamformed in-phase and quadrature (IQ) data and envelope detected image data can also be accessed. This framework is illustrated in Figure 1.

The Verasonics software package provides example programming scripts for commonly used imaging sequences, such as plane wave and focused transmit B-mode sequences. In addition, it offers example scripts for shear wave imaging for the L7-4 linear array in recent software releases (v2.10 and later) for Vantage 128 and 256 systems. These shear wave imaging example scripts provide templates for making MTL-SWEI sequences with a single push at the center of the transducer, plane wave tracking, and using the Verasonics beamforming to produce beamformed IQ data. Given the availability of the example scripts, this section focuses on the parameter selection for SWEI sequences, including guidelines of setting important SWEI parameters and the specific MATLAB structures that contain each parameter in the programming script. We assume the audience has basic knowledge of Verasonics sequencing, and will frequently refer to the example script for the L7-4 linear array “ setupl7_4shearwaveimaging.m” from the latest Verasonics software version 3.0.6 as an example.

In addition, we provide an example script, “ setupc5_2shear_wave_mtl.m”, which is a MTL-SWEI sequence for the C5-2 curvilinear array. This C5-2 script can serve as a template for researchers interested in using shear wave techniques in abdominal applications, such as in liver fibrosis staging [3]. It can be found in a publicly available Github repository at https://github.com/yufengdeng/Verasonics_ARFI/tree/1.0 (DOI: 10.5281/zenodo.59619). When this script is executed, the Verasonics system enters real-time B-mode imaging mode, with a B-mode image shown on the computer screen. When the user clicks the B-mode image on the screen, the sequence then enters shear wave imaging mode and executes the MTL-SWEI sequence. We will also refer to this C5-2 example script in the following sections.

Table II lists the SWEI parameters considered in this paper and the names of the MATLAB structures that contain these parameters in the Verasonics programming script. The detailed descriptions of these structure variables can be found in the Verasonics user manual.

### A. Push and track transmit frequencies

The push and track frequencies are defined in separate transmit waveform ( tw) structures. The track transmit frequency is typically selected to be equal to the center frequency of the transducer, which can be found in the structure trans.frequency. A lower track transmit frequency is generally used in the case of harmonic tracking, to enable the transducer to effectively receive at the harmonic frequency.

The push transmit frequency is nominally set to the center frequency of the transducer to allow maximum transmit efficiency to deliver acoustic radiation force to the tissue; however, it is often preferable to use a lower push frequency to broaden the push beam width compared to the track beam width to reduce the underestimation of tracked tissue displacement due to speckle shearing within the track point spread function (PSF) [14], [15]. Our general approach is to use a push frequency that is within the lower -6 dB bandwidth of the transducer to broaden the push beam while maintaining high transmit efficiency.

In the example scripts, tw(1) and tw(2) are used to define the track and push waveforms respectively. The transmit frequencies are found in the first variable of the vector tw.parameters. For the Verasonics L7-4 example script, the transmit frequency of both push and track is set to the center frequency of the transducer, which is 5.2 MHz for the L7-4 transducer. In our C5-2 script, the track frequency is set to 3.1 MHz, which is the center frequency of the C5-2 transducer. The push frequency is set to 2.4 MHz, which is at the lower edge of the -6dB bandwidth. This frequency was determined by measuring the impulse response of the transducer.

### B. Push duration

The force density (*f*) delivered by an acoustic beam can be expressed in terms of the pulse-averaged acoustic intensity (*I*) and the ultrasonic attenuation (*α*) of the tissue as [16]

(2) $$\vec{f}=\frac{2\alpha \vec{I}}{c},$$

where *c* is the sound speed in the tissue. For an impulsive excitation with duration *T*, the mechanical impulse delivered to the tissue is given by the product *fT*, and the tissue displacement magnitude is, in turn, proportional to this mechanical impulse. On the Verasonics scanner, the duration *T* of the applied excitation is determined by the number of cycles of the push waveform in the tw structure. Therefore, increasing the push duration will increase tissue displacement, which can result in higher shear wave signal-to-noise ratio (SNR) and higher likelihood of successful SWS estimation [17]. However, it is possible to experience scanner power supply droop over a long duration push pulse, depending on the power supply characteristics. The pressure amplitude gradually decays towards the end of the push pulse when the scanner power supply is depleted. In addition, it has been shown that the push duration has an impact on the shear wave spectral content [18]. Increasing the push duration can result in a decrease in shear wave center frequency, which can lead to a change in the group SWS measured in viscoelastic materials. In setupl7_4shearwaveimaging.m, the default push duration is set to 1000 cycles, which corresponds to a duration of 192 *μ*s at 5.2 MHz. In setupc5_2shear_wave_mtl.m, the push duration is set to 800 cycles, which corresponds to a duration of 333 *μ*s at 2.4 MHz.

### C. Push and track transmit aperture and focal configurations

The transmit aperture and focal configurations are defined in the tx structures. Specifically, the transmit aperture location is defined by tx.origin, the focal depth is defined by tx.focus, and the transmit F-number is determined by the apodization settings in tx.apod. The push and track transmit focal configurations are heavily dependent on the type of the imaging sequence. In setupl7_4shearwaveimaging.m, where an MTL-SWEI sequence is made, plane wave tracking is used to insonify the entire field of view (FOV) for each transmit to allow shear wave propagation to be monitored throughout the FOV. However, this large FOV results in sub-optimal data quality compared to focused tracking. The tracking configuration is defined in tx(1), where tx(1).focus is set to 0, which corresponds to plane wave (no transmit delays) for the tracking pulses. All elements are used in plane wave tracking pulses, set by tx(1).apod. In addition, a single push originates from the center of the transducer to generate the ARFI excitation. The push beam configuration is defined in tx(2), where tx(2).focus is set by default to 50 wavelengths, which corresponds to 14.8 mm at a frequency of 5.2 MHz. The center 32 elements of the transducer are active for the push beam as specified by tx(2).apod, which corresponds to an F/1.5 focal configuration (F = *z*/*D*, *z* = 14.8 mm, *D* = 0.3×32 = 9.6 mm). Our C5-2 script setupc5_2shear_wave_mtl.mis configured in a similar way. The track transmit has zero delays across the transducer elements, resulting in a diverging wave due to the curved transducer surface. The push transmit has an F/2 focal configuration at 50 mm.

It has been shown that SWS measurements at depths away from the elevational lens focus of the transducer can lead to over-estimation of SWS using time-of-flight methods, which has been attributed to out-of-plane shear wave sources [19]. For accurate SWS measurements, it is recommended to set the lateral push focus concurrent with the elevational focus of the transducer. The elevational foci of the L7-4 and C5-2 transducers are reported to be 25 mm [19] and 55 mm [20] respectively.

### D. Push and track receive configurations

Ultrasound elasticity imaging sequences typically perform tissue motion tracking using repeated transmit and receive tracking events at the same spatial locations. A single tx structure can often be used to describe the identical transmit characteristics for these tracking events. However, each repeated receive event has to be described by a unique receive structure, which contains the unique memory location of the received channel data. In setupl7_4shearwaveimaging.m, tx(1) is used to describe all the tracking transmits. Each tracking receive event has its corresponding Receive structure entry, with a unique combination of the variables receive.bufnum, receive.framenum, and receive.acqnum, which specify the memory location of the received channel data. The Receive structure definitions work in conjunction with the rcvbuffer structure definitions in the programming script to correctly allocate memory space for the channel data on the host computer.

### E. Sampling frequencies

The sampling frequency information of the channel data is typically required for post-processing. In Verasonics software version 3.0 and beyond, the channel data sampling frequency is defined in receive.samplemode, which is set to “NS200BW” by default. This means that the sampling frequency is 4 times the center frequency of the transducer, which can be found in trans.frequency. In the previous software versions 2.x, the channel data sampling frequency is defined in receive.samplesperwave, which is set to 4 by default corresponding to a sample mode of “NS200BW”.

The spatial sampling intervals of the beamformed IQ data using Verasonics beamforming are defined in the pdata.pdelta structure. The user is able to specify different sampling intervals in the x,y,z (lateral, elevational, axial) dimensions. In the L7-4 example script, pdata(2) contains the beamforming characteristics for the SWEI data. Since the L7-4 is a linear array, the sampling interval is only defined in x and z dimensions. The axial sampling interval is set to 0.25, which means that the axial spacing interval of the IQ data is 1/4 of the center wavelength, which corresponds to an axial sampling interval of 74 *μ*m (= 0.25 × 1540 m/s ÷ 5.2 MHz). The default lateral sampling interval is equal to 0.5, which is half of the center wavelength. In our C5-2 example script, the lateral and axial sampling intervals are also set to 0.5 and 0.25 respectively, corresponding to 246 *μ*m and 123 *μ*m at 3.1 MHz. In previous Verasonics software versions 2.x, the lateral and axial sampling intervals are defined in structures pdata.pdeltax and pdata.pdeltaz respectively.

### F. Pulse repetition interval (PRI)

The PRI determines the sampling rate used to track shear wave propagation and determines the maximum shear wave speed that can be tracked due to the Nyquist sampling criterion. The PRI is specified in SeqControl structure by timetonextacq, in terms of microseconds. The PRI needs to be sufficiently large for the sound wave to travel through tissue and scatter back to the transducer between tracking frames. If the PRI specified is too small, a warning message will be displayed in the MATLAB command window during runtime. In setupl7_4shearwaveimaging.m, the PRI between tracking frames is set in seqcontrol(6) to 100 *μ*s, corresponding to a pulse repetition frequency (PRF) of 10 kHz. In addition, the time between the push and the first track frame is set in seqcontrol(5) to 500 *μ*s, to account for the extended push pulse. In setupc5_2shear_wave_mtl.m, the PRI between tracking frames is set in seqcontrol(8) to 200 *μ*s, corresponding to a pulse repetition frequency (PRF) of 5 kHz. The time between the push and the first track frame is set in seqcontrol(10) to 400 *μ*s.

### G. Push and track excitation voltages

The operating push and track excitation voltages are by default specified during runtime in the imaging GUI window. The maximum voltage limit allowed in the GUI is defined in the programming script by trans.maxhighvoltage. Increasing the push voltage will increase tissue displacement, as a higher push voltage leads to a higher intensity of the push beam in (2). Increasing the track voltage will lead to a higher SNR of the tracking data, which can increase SWS estimation yield. It is noted that the push and track use the same voltage, as they use the same power supply on the system. The L7-4 example script limits the maximum voltage limit to be 65 V, to protect the transducer from overheating when using higher voltages. This limit can vary significantly for different transducers. Based on our experience, we set the maximum voltage limit of the C5-2 transducer to 75 V.

## III. Calibration of beam positions and scanner timing

### A. Beam position calibration

Several research groups have interfaced the Verasonics system with commercial transducers [19] and custom made transducers [11], for which control scripts are not available in the Verasonics software. In our experience, the calibration of transducer parameters in research systems is likely to be less rigorous than in commercial systems. In many cases, the exact transducer geometry parameters might not be known to the researcher, particularly in the case of commercial transducers when these parameters represent proprietary information. Incorrect transducer parameters lead to biases in SWS measurements [21]. More specifically, pitch errors in linear and phased arrays, or radius of curvature (ROC) and sector angle errors in curvilinear arrays, lead to delay computation errors in the beamforming process. Incorrect delays in turn result in beam position errors, as well as a de-focused ultrasound image. Errors in lateral beam position result in errors in SWS estimation. Therefore, it is important to calibrate the lateral beam position for an imaging system to avoid the SWS estimation bias.

**Fig. 2:**
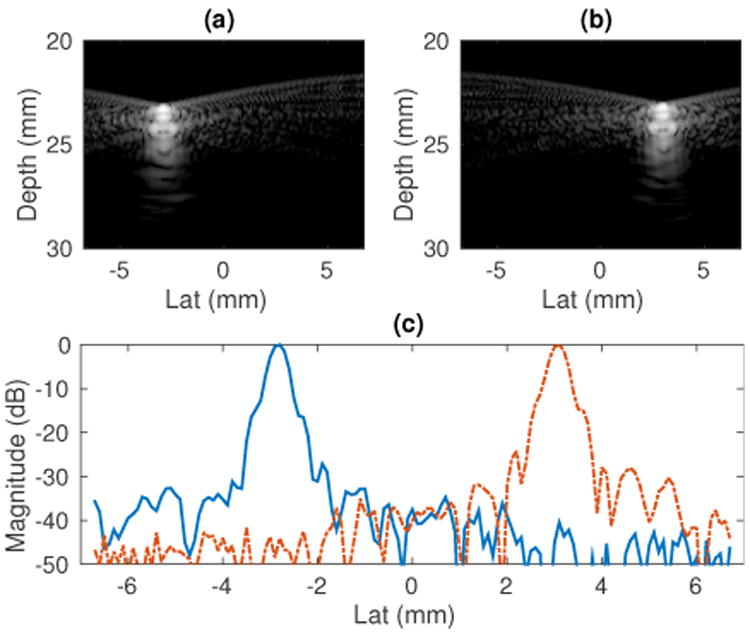
Calibration of beam positions by imaging point targets with known lateral translations. (a) and (b): images of the point target using the L7-4 linear array. The point target was moved laterally by 6.0 mm using a translation stage between the acquisitions of the two images. (c): lateral PSF signals of the point targets. The cross-correlation of the lateral PSF signals gave a lateral translation of 5.8 mm, indicating a pitch error of 3%.

The calibration of beam positions can be accomplished by imaging point targets with known lateral translations. The recommended equipment are a point target (wire target), water tank, and a translation stage. The procedure is summarized as follows:

1. Submerge the point target in water.
2. Measure the speed of sound in water [22], and input the correct speed of sound in the Verasonics programming script.
3. Mount the transducer to a translation stage with < 0.01 mm precision, and make an image of the point target (Figure 2(a)).
4. Laterally translate the transducer by a prescribed distance (several millimeters) using the translation stage, and make another image of the point target (Figure 2(b)).
5. Perform cross-correlation between the images of the point target, and obtain the lateral translation of the target between the images from the maximum correlation value.
6. Compare the lateral translation between the images and translation stage reading.

There are several points to note when carrying out these procedures.

- A Bmode calibration phantom consisting of point targets can be used instead of the point target in the water tank, provided that the speed of sound in the phantom is known. It is important to input the correct speed of sound in the Verasonics programming script.
- For linear arrays and phased arrays, the point target can be located at any axial depth. A pitch error would result in the same lateral beam position errors at all depths [21].
- For phased arrays, it is recommended to image the point target close to the center of the array to minimize errors introduced by steering. A percent pitch error causes the same percent error in lateral beam position error with no steering, but this percent beam position error changes as steering angle increases [21].
- For curvilinear arrays, it is useful to perform the calibration at a series of axial depths. An ROC error causes different lateral position errors at different depths, while a sector angle error results in the same lateral beam position error at all depths [21].
- It is recommended to upsample the image data laterally before the cross-correlation calculation in step (5) to increase the resolution of the correlation shift. We recommend a lateral sampling interval of ≤ 0.1 mm.

Figure 2(a) and (b) show an example pair of images on the point target using the L7-4 linear array. The point target was moved laterally by 6.0 mm using a translation stage between the acquisitions of the two images. Figure 2(c) shows the lateral PSF signal of the point targets. The cross-correlation of the lateral PSF signals gave a lateral translation of 5.8 mm, which corresponds to a 3.33% lateral position error. The 3.33% lateral beam position error directly translates to a 3.33% pitch error [21]. The translation stage used in this experiment has a precision of 0.001 mm, which could introduce a mechanical error of 0.03% to the lateral movement reading of the point target, and therefore results in a 0.03% uncertainty in the pitch error measured. The pitch error is (3.33 ± 0.03)%. The transducer pitch can then be corrected in computetrans.m, which contains the transducer parameters in the Verasonics software.

### B. Scanner timing calibration

It is also important to calibrate for the scanner timing to ensure that the PRI used by the system is equal to the PRI set in the programming script. Using the Verasonics system, the user is able to set up a trigger output at every transmit event, and visualize the trigger signal using an oscilloscope. It is important to ensure that the time between each trigger signal is equal to the PRI determined in the programming script. A percent error in PRI would translate to the same percent error in the SWS estimate [21]. The trigger output of the Verasonics system can be defined in the seqcontrol structure in the programming script, with the command triggerout. In both setupl7_4shearwaveimaging.m and setupc5_2shear_wave_mtl.m, the trigger output is defined in seqcontrol(9), and a trigger signal is attached to every push transmit.

Transducer parameter errors and PRI errors are system dependent sources of bias in a SWS measurement. Calibration of these sources of error is an important step in the development of shear-wave imaging systems to avoid SWS measurement bias. In a well-calibrated system, other sources of uncertainty such as speckle bias and phase aberration can result in variations in SWS measurements. In a typical well-calibrated system for liver fibrosis staging, system-dependent sources lead to <3% errors in SWS measurements [21].

## IV. Avoiding transducer damage

It is possible to damage the transducer running SWEI sequences with high voltages on the Verasonics system, particularly with the extended high-intensity-focused-ultrasound (HIFU) power supply. The long-duration push pulses in the SWEI sequences can lead to overheating of the lens, and eventually result in de-lamination of the lens as well as irreversible transducer damage. To avoid such damage, we recommend performing transducer face heating measurements of SWEI sequences according to the International Electrotechnical Commission (IEC) standard [23] under test conditions 201.11.1.3.1.1, where the transducer is coupled with a tissue mimicking phantom. It is not recommended to perform the measurements while transmitting into air under test conditions 201.11.1.3.1.2, which can lead to rapid heating of the transducer due to the reflection of the long-duration push pulses at the transducer/air interface. The IEC standard requires that all transducers must operate such that the temperature at the surface, when coupled to tissue, can never exceed 43°C, allowing a 6°C rise from body temperature [23].

We also recommend running the same sequence on a calibration phantom regularly to assess displacement amplitude. The transducer is damaged if the displacement amplitude decreases over time. This damage can be a gradual process and can be difficult to detect from B-mode images. In addition, during the process of sequence development, we recommend decreasing the number of cycles in the push pulse and using a small excitation voltage if possible to minimize the risk of transducer damage.

## V. Translation to clinical research

The Verasonics system is not marketed for clinical use. If a SWEI sequence is intended for clinical research, we recommend that research groups work with their Institutional Review Board (IRB) to obtain approval to use the Verasonics system on human subjects. Additional measurements are required to ensure that the acoustic and thermal output of the sequences are within the Food and Drug Administration (FDA) and American Institute of Ultrasound in Medicine (AIUM) output guidelines [24]. Please refer to the AIUM/NEMA [25] standard for the procedures of ultrasonic acoustic and thermal output measurements prior to clinical imaging.

It is noted that the provided L7-4 and C5-2 sequencing example scripts have not undergone acoustic output and transducer face heating measurements. The users are advised to perform these measurements to ensure the safety of their imaging sequences and to document and report them to their IRB during the process of obtaining IRB approval prior to considering performing clinical research with these sequences.

## VI. Data processing

This section describes the processing pipeline to calculate the group SWS from data acquired in a SWEI sequence. This processing pipeline involves 3 major steps: (1) tissue motion estimation, (2) data filtering, and (3) SWS estimation. We will demonstrate the basic procedures for each step, assuming that the raw data is of high quality taken from a homogeneous elastic phantom. In addition, we will introduce other commonly used algorithms that deal with more challenging datasets.

### A. Tissue motion estimation

There are many time-delay estimators available for tissue motion tracking. They can be broadly characterized into 2 categories [26]: correlation based approaches that operate on RF data, and phase-shift algorithms that operate on IQ data. Using a Verasonics system, the user is able to access the raw channel RF data, the beamformed IQ data, and the envelope detected image data. Envelope detected image data lacks the phase information needed to track micron scale displacements typical in ultrasound SWEI. We will describe a commonly used phase-shift algorithm, the Kasai algorithm [27], which can be directly applied to the beamformed IQ data out of the Verasonics system.

#### 1) Kasai algorithm

The Kasai algorithm [27], also known as the 1-D autocorrelator, is commonly used in tissue displacement estimation using beamformed IQ data. This algorithm is included in the Verasonics example script setupl7_4shearwaveimaging.m. The Kasai algorithm was originally developed for blood flow velocity estimation in Doppler systems and determines the displacement between a reference and displaced signal by measuring the average phase shift with respect to the center frequency. This algorithm calculates the average displacement *ū* over an axial sample range *M* and ensemble length *N* using (3) [26], where *c* is the speed of sound in tissue, *f_c_* is the center frequency of the RF data before demodulation, and *I* and *Q* are the in-phase and quadrature components of the IQ data. This phase-shift algorithm only estimates displacements in the range −*λ*/2 ≤ *ū ≤ λ*/2.

#### 2) Fixed and progressive referencing

In the formulation of (3), the signal at each time step is compared with the previous time step, and the tissue displacements are averaged over the ensemble length *N.* This procedure is commonly used in Doppler imaging, where the tracking beams are repeated *N* times at the same location to produce a single velocity estimate. For shear wave imaging, the signal trace at a fixed time step before the push is typically used as the reference signal, and the tracking data at each time step after the push are compared to the reference signal to calculate the displacement at each time step. This is called a fixed reference scheme. For example, if the data at time *n* = 0 is used as the reference, the average displacement *ū_i_* at time *t_i_,* relative to the fixed reference, is given by (4).

Alternatively, in a progressive reference scheme, each IQ signal trace is compared to the signal at the previous time step so that the differential average displacement Δ*ū_i_* at each time step is calculated given by (5). The axial velocity signal can be calculated from these displacements by dividing by the time step interval Δ*t*,

(6) $${\bar{\upsilon }}_{i}=\frac{\Delta {\bar{u}}_{i}}{\Delta t}$$

If needed, the tissue displacement signal can be calculated by accumulating the differential displacements.

Compared to the fixed reference procedure, the progressive reference procedure is preferred in cases with large (> *λ*/2) accumulated displacements or cases with large speckle decorrelation when the reference and track signals are separated by large time intervals.

#### 3) Other phase-shift algorithms

The Loupas 2-D autocorrelator [28] is another commonly used displacement estimation algorithm. It is an extension of the Kasai algorithm, and uses information from the depth samples within an axial range to calculate displacement. The difference between the Kasai and Loupas algorithms is that the Kasai algorithm assumes a constant carrier frequency for the data while the Loupas algorithm calculates the mean Doppler frequency and the mean RF frequency at each axial position. In other words, the Loupas algorithm corrects for local variation of the center frequency and has been shown to yield more accurate displacement estimates [26]. In addition, iterative algorithms have been described that aim to be robust with noisy data. For example, Pesavento *et al.* [29] implemented a real-time, iterative algorithm that uses phase zero seeking to calculate displacement, and Byram *et al.* [30], [31] described a Bayesian displacement estimator.

#### 4) Correlation based algorithms

Using the Verasonics system, the beamformed IQ data can be re-modulated to create RF data, or by operating on the raw channel data, custom beamforming schemes can be used to create RF data. Then, conventional correlation based methods such as normalized cross-correlation (NXcorr) [26] can be used for displacement estimation. As pointed out in [26], with a sampling frequency of 40 MHz, an axial displacement of one sample is approximately 20 *μ*m, which is generally larger than ARFI induced displacement. Therefore, it is necessary to upsample the RF data before NXcorr to increase the ability to resolve small displacements. Pinton *et al.* [26] used a combination of cubic spline interpolation and parabolic fitting to the maximum of the correlation function. Correlation based methods are significantly more computationally intensive than phase-shift algorithms, so that the latter are more readily used in real-time applications.

### B. Data filtering

After tissue motion data are obtained, several steps are typically performed to reduce the noise in the data before SWS estimation. These steps include: (1) removal of reverberation frames, (2) low-pass filtering (LPF) to reduce jitter, and (3) motion filtering and directional filtering.

#### 1) Removal of reverberation frames

It is usually difficult to track tissue motion accurately in the first few track frames immediately after the push because the displacement estimation at these time steps often have very high spatial variations and unrealistic amplitudes. It is hypothesized that this phenomenon is caused by the inability to track displacements in the presence of clutter from the push pulse, arising from the reverberation of the long duration push pulse in the field. Tracking at a larger lateral distance from the push beam and using a longer

(3) $$\bar{u}=\frac{c}{4\pi f_{c}}\text{arctan}(\frac{\sum_{n=0}^{N-2}[\sum_{m=0}^{M-1}Q(m,n)\sum_{m=0}^{M-1}I(m,n+1)-\sum_{m=0}^{M-1}I(m,n)\sum_{m=0}^{M-1}Q(m,n+1)]}{\sum_{n=0}^{N-2}[\sum_{m=0}^{M-1}I(m,n)\sum_{m=0}^{M-1}I(m,n+1)+\sum_{m=0}^{M-1}Q(m,n)\sum_{m=0}^{M-1}Q(m,n+1)]})$$

(4) $${\bar{u}}_{i}=\frac{c}{4\pi f_{c}}\text{arctan}(\frac{\sum_{m=0}^{M-1}Q(m,0)\sum_{m=0}^{M-1}I(m,i)-\sum_{m=0}^{M-1}I(m,0)\sum_{m=0}^{M-1}Q(m,i)}{\sum_{m=0}^{M-1}I(m,0)\sum_{m=0}^{M-1}I(m,i)+\sum_{m=0}^{M-1}Q(m,0)\sum_{m=0}^{M-1}Q(m,i)})$$

(5) $$\Delta {\bar{u}}_{i}=\frac{c}{4\pi f_{c}}\text{arctan}(\frac{\sum_{m=0}^{M-1}Q(m,i-1)\sum_{m=0}^{M-1}I(m,i)-\sum_{m=0}^{M-1}I(m,i-1)\sum_{m=0}^{M-1}Q(m,i)}{\sum_{m=0}^{M-1}I(m,i-1)\sum_{m=0}^{M-1}I(m,i)-\sum_{m=0}^{M-1}Q(m,i-1)\sum_{m=0}^{M-1}Q(m,i)})$$

PRI help to reduce the number of these reverberation frames. However, tracking far away from the push could lead to smaller displacement amplitudes and lower SNR, and a longer PRI limits the maximum SWS that can be tracked with the sequence.

**Fig. 3:**
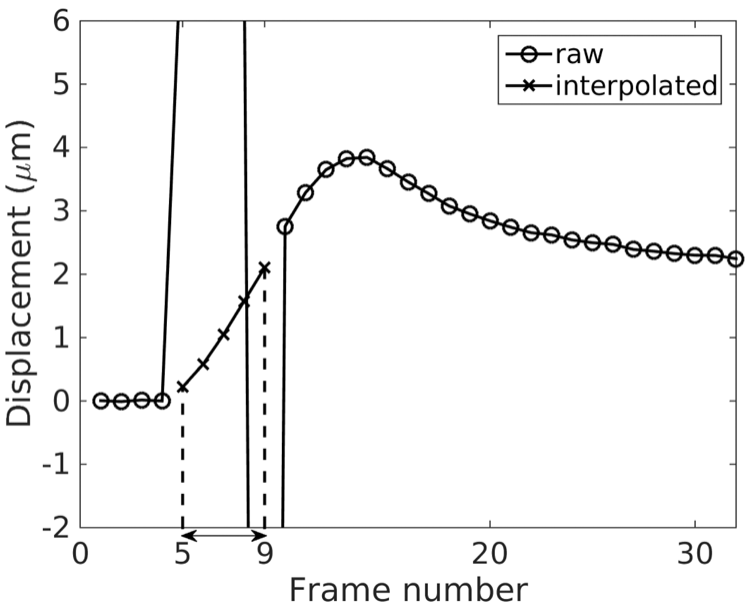
Typical experimental ARFI displacement profile over time. The displacements in frames 5-9 have unrealistic amplitudes extending beyond the vertical axis limit. The displacements at these 5 frames (3 push frames, 2 reverberation frames) are reconstructed by cubic spline interpolation. The peak particle velocity signal occurs in these 5 interpolated frames. The PRI was 0.2 ms.

A typical approach is to remove these reverberation frames from the tissue motion profile after the data is collected. If reference data is recorded before the push, motion data can be interpolated in the slow time dimension (the time dimension along the repeated tracking frames) to reconstruct the data at those missing frames. This interpolation step helps to maintain uniform temporal sampling of the motion data so that downstream imaging processing techniques such as LPFs can be easily implemented. For example, Figure 3 shows a typical experimental displacement profile over time from a homogeneous elastic phantom. There are 4 reference frames, 3 push frames and 25 track frames. 5 frames of raw displacements have unrealistic amplitudes (frames 5-9), 3 of them are due to push frames and the other 2 are due to reverberation frames. Cubic-spline interpolation was performed to reconstruct the displacement profiles for the 5 time steps to achieve uniform temporal sampling. Alternatively, these data, in addition to the reference time steps before the push, can be removed.

#### 2) Low-pass filtering (LPF) to reduce jitter

Signal decorrelation, finite window lengths, and noise can result in random errors in the tissue motion data. After the reverberation frames are removed, a LPF is typically applied to the tissue motion data to reduce the high frequency jitter. The cut-off frequency of the LPF has to be chosen high enough so that shear wave energy is not rejected by the filter. We recommend a cut-off frequency of 1500 Hz for the displacement data collected with a L7-4 transducer. We also recommend to perform a Fourier transform on the tissue motion data to check the distribution of the shear wave energy in the frequency domain, and choose the cut-off frequency for the LPF accordingly.

#### 3) Motion filtering and directional filtering

In more challenging scenarios, motion filters and directional filters can be used to reduce background motion and waves reflected from boundaries. Motion filters are typically applied to clinical data to reduce the background motion arising from biological motion and transducer motion. A high pass filter (HPF) can serve as a motion filter because biological motion is generally at low frequency (< 10 Hz), while the ARFI generated shear waves typically have a center frequency greater than 50 Hz. It is noted that the HPF rejects the DC component of the tissue displacement data, forcing the average signal to be zero. This effect decreases the maximum signal, but is not a problem in shear wave imaging, as long as the shape of the displacement signal over time is preserved. Alternatively, using particle velocity data *υ*(*t*), or differentiating the displacement data to create the particle velocity data as in (6), is less sensitive to low frequency motion because of its higher frequency content. Higher frequencies are weighted more in the velocity data as described by the Fourier transform relation

(7) $$\begin{array}{c} \upsilon (t)=\frac{du}{dt}\\ F[\upsilon (t)]=i\omega F[u(t)]. \end{array}$$

In addition, a directional filter can be applied to reduce the artifacts from reflected shear waves at boundaries [32]. When shear waves propagate through an interface where there is a change in the stiffness, reflections will occur and the reflected waves can lead to artifacts in SWS reconstruction. Deffieux *et al.* [32] described a 2D directional filter in Fourier (*k_x_*, *ω*) space to separate leftward and rightward traveling waves. Song *et al.* [7] described a similar approach in the CUSE technique. Higher dimensional directional filters were recently proposed to deal with in-plane and out-of-plane interfaces [33], [34].

#### 4) Displacement and velocity data

Both particle displacement and particle velocity data can be used for SWS estimation. It is possible to convert between displacement and velocity data. In the tissue motion estimation step, tissue displacement *ū* is obtained by default if using a fixed reference scheme. Particle velocity *ῡ_i_* at time step *i* can be obtained by differentiating the displacement data with respect to the slow time:

(8) $${\bar{\upsilon }}_{i}=\frac{d{\bar{u}}_{i}}{dt}{|}_{t=t_{i}}$$

On the other hand, if a progressive reference scheme is used, particle velocity *ῡ* is obtained after dividing differential displacement Δ*ῡ* by the sampling time interval Δ*t*. The absolute displacement at time step *i* can be obtained by accumulating the differential displacements up to that time step,

(9) $${\bar{u}}_{i}=\sum_{j=1}^{i}\Delta {\bar{u}}_{j}=\Delta t\sum_{j=1}^{i}\Delta {\bar{\upsilon }}_{j}.$$

Particle velocity data is generally favored to be used in SWS estimation. It is less susceptible to wave shape changes introduced by the reflections at the boundaries and background motion [35]. However, the peak of the particle velocity occurs earlier in time compared to the peak of displacement, making the former more difficult to detect properly in the presence of reverberation frames or in the case of stiff materials. For example, in the case shown in Figure 3, the maximum displacement can be easily extracted from the raw data, while the peak particle velocity occurs during the reverberation frames. Therefore, the peak particle velocity signal has to be reconstructed using interpolation which can introduce bias and uncertainties depending on the interpolation scheme.

As indicated in (7) above, the shear wave signal reconstructed from particle velocity data has a higher frequency content than the shear wave reconstructed by displacement data. In the case of a viscoelastic medium where shear wave dispersion occurs, displacement and particle velocity data generally result in different group SWS estimates.

### C. Group SWS estimation

Ultrasonic displacement estimation is subject to noise, and SWS estimation approaches based on direct inversion of the wave equation [36] requiring second-order spatial and temporal derivatives generally fail [6], [37]. As a result, time-of-flight (TOF) methods are typically used in ultrasonic SWS estimation. TOF methods take advantage of *a priori* information about the shear wave propagation direction to estimate wave speed [38], [39]. In 2D shear wave imaging, the ARFI push displaces tissue in the axial direction, and the shear wave is assumed to travel in the lateral direction. There are 2 types of TOF methods that are widely used in SWS estimation: linear regression and shear wave trajectory detection.

#### 1) Linear regression

**Fig. 4:**
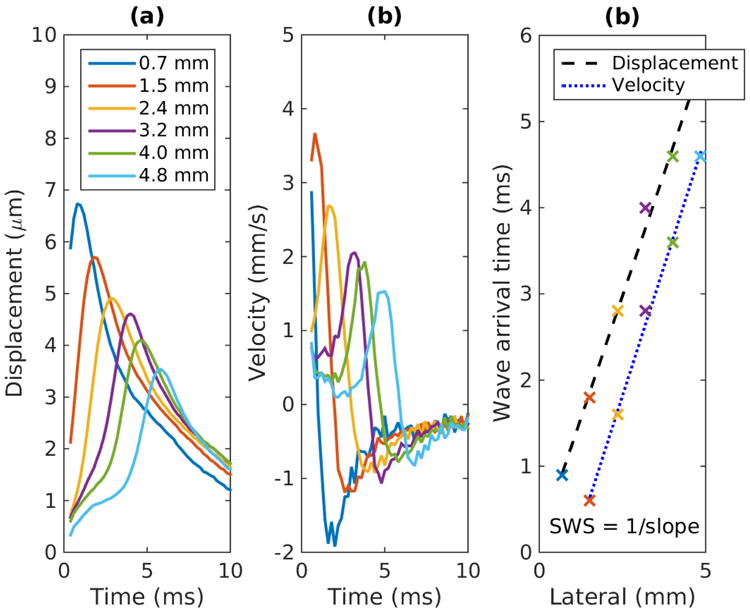
Linear regression on wave arrival time profile. (a) and (b) Experimental particle displacement and velocity versus time profiles at the focal depth at several lateral locations resulting from an ARFI excitation at lateral position of 0 mm measured on a homogeneous elastic phantom. The legend in (a) indicates the lateral position of each displacement trace. (c) Shear wave arrival time versus lateral distance from excitation. Arrival times were estimated from the time of peak displacement and peak velocity. The dashed and dotted lines show the linear fits. For both cases, the SWS is equal to 1.1 m/s.

Linear regression is widely used as a TOF method to calculate SWS. This approach first determines the shear wave arrival time at each lateral position, and then performs a linear regression on wave arrival time versus lateral position data to calculate SWS. The wave arrival time can be calculated from the tissue motion data using several characteristic features of the shear waves including time of peak displacement [38], time of peak particle velocity [35], and cross-correlation of tissue motion signal at adjacent spatial locations [39]. The SWS is given by the slope of the linear regression between wave arrival times and lateral positions. Figure 4 demonstrates this method using an example set of experimental data collected in an elastic phantom. Figure 4(a) and (b) show the particle displacement and velocity profiles at 6 lateral positions. Figure 4(c) plots the wave arrival times calculated from the time of peak displacement and the time of peak velocity. Linear regressions using least square minimization were performed on arrival times versus lateral positions, and the linear fits with least square error are shown. SWS is equal to the inverse of the slope of the linear fits in Figure 4(c). Least square regression methods are sensitive to outlier data. It is recommended to use a large number of lateral positions (≥6 lateral positions covering several millimeters) in the lateral region of interest (ROI) in the linear regression to reduce the impact of any particular outlier data point.

Compared to least square minimization, other forms of linear regression are less sensitive to outlier data. Wang *et al.* [40] applied the RANdom Sample Consensus (RANSAC) algorithm [41] to the problem of SWS estimation. In this approach, a trial solution is calculated by randomly selecting a minimal set of data points, and then enlarging this data set by including all other data consistent with this solution. The optimal SWS estimate is found by iterating on the trial solutions and selecting the solution with the greatest number of consistent data. The RANSAC algorithm was proven to be robust in the presence of outlier data, and can provide the percentage of inlier data points, which can be used for quality control. However, RANSAC requires a large number of iterations on trial solutions to have a high probability of finding the optimal SWS estimate. Wang *et al.* used 5000 trial solutions in [40], which was not suitable for real-time applications.

#### 2) Shear wave trajectory detection [42]

**Fig. 5:**
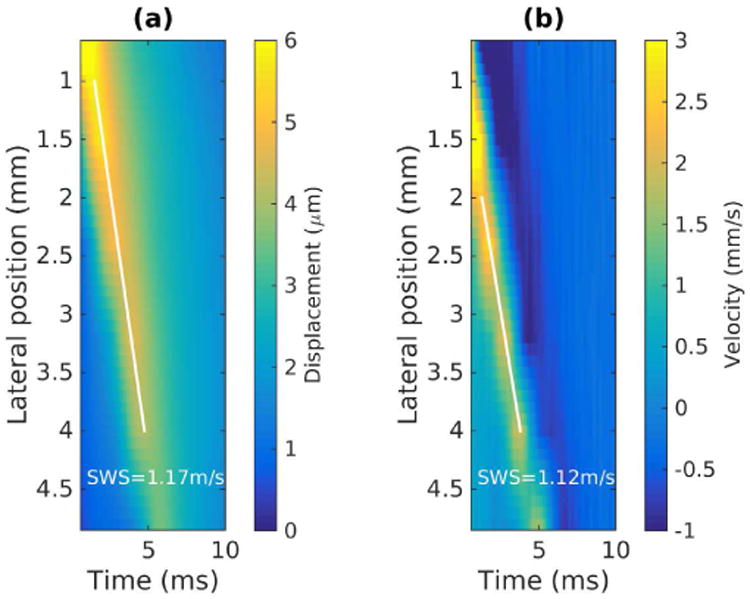
Shear wave trajectory detection by Radon sum transformation. (a) Displacement data from Figure 4(a) is displayed as a two-dimensional image in lateral position and time. The white line indicates the optimal shear wave trajectory identified by the peak Radon sum. The lateral extent is restricted in 1 - 4 mm in the calculations of Radon sums. (b) The same procedure was repeated on velocity data, where the lateral extent is restricted in 2 - 4 mm in the calculations of Radon sums. The track locations less than 2 mm from the push did not capture the peak particle velocity, and therefore did not provide accurate wave arrival times.

Another commonly used TOF technique to estimate SWS is to identify the spatial-temporal shear wave trajectory from the shear wave signal expressed as a function of the position and time coordinates. The shear wave motion can be characterized by the particle displacement or particle velocity signals, and the SWS is equal to the slope of spatial-temporal trajectory with the maximum energy. Figure 5 shows an example of this method using the same data set used for the data shown in Figure 4(a). The wave trajectory is shown by the white line obtained using the Radon sum transformation. As implemented by Rouze *et al.* [42], this approach considers a solution space of linear trajectories extending from a fixed starting position and multiple starting times, to a fixed ending position and multiple ending times. For each starting and ending point, the shear wave signal is integrated along the trajectory in the same way that projection data is calculated in the Radon transformation of an image. The optimal trajectory is identified by the peak Radon sum and is indicated by the white line in Figure 5. SWS is calculated from the slope of this trajectory. It is noted that Rouze *et al.* upsampled the 2D motion data in the time dimension to increase the time resolution of this algorithm [42].

#### 3) Axial averaging and lateral windowing

The SWS estimation algorithms described above expect 2-D input tissue motion data (lateral positions × time). In our laboratory, we typically project the raw 3-D (axial × lateral × time) tissue motion data into 2-D data (lateral × time) by averaging over the axial depth of field (DOF) defined by 8*F*^2^*λ* around the focal depth of the push beam, where *F* is the F-number and *λ* is the wavelength of the push beam. The TOF SWS estimation methods assume the shear wave travels in the lateral direction, which is most valid within the DOF. Alternatively, it is not necessary to average over the full DOF. Many groups use a smaller, fixed depth, to increase the axial resolution of the SWS estimate.

SWEI sequences with a lateral push focus away from the elevational lens focus of the transducer can lead to over-estimation of SWS using TOF methods [19]. Separate lateral and elevational push foci can result in a undesired intensity field distribution of the push beam that generates out-of-plane shear wave sources. The shear waves originating from the out-of-plane sources travel into the axial/lateral plane of interest and cause SWS estimation bias. As described in Section II C, it is recommended to set the lateral push focus to agree with the elevational focus of the transducer during SWEI sequencing.

**Fig. 6:**
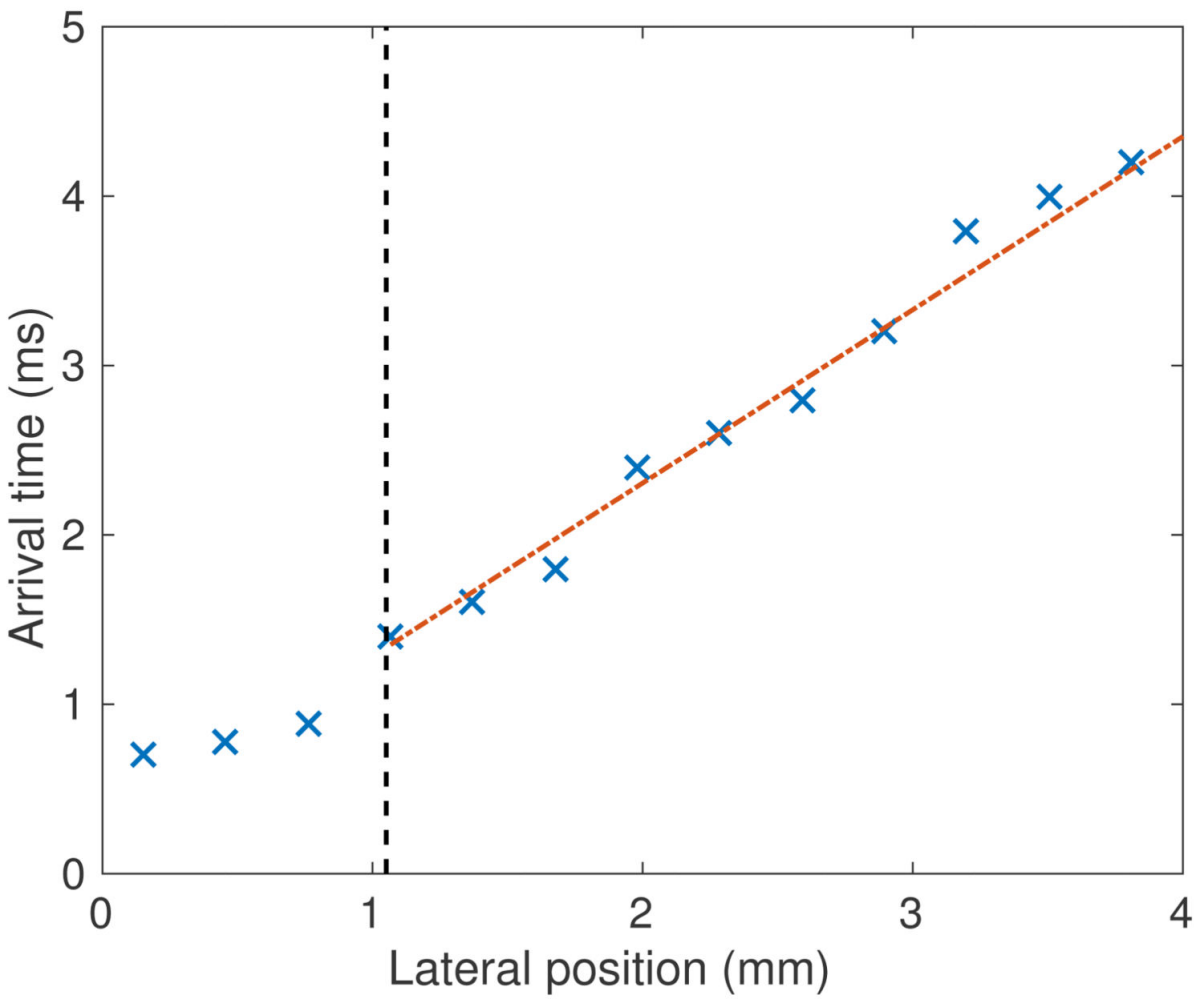
Wave arrival times versus lateral positions from experimental data measured on a homogeneous elastic phantom. The push was at 2.2 MHz with an F/1.5 focal configuration, and located at 0 mm. The black dashed line shows the boundary of the excitation beam. The wave arrival times at the first 3 lateral positions within the excitation beam width are inconsistent with the linear trend between the arrival times and positions measured at other lateral positions beyond the excitation beam width.

Another source of bias in SWS estimation arises when the lateral regression window starts within the near-field of the shear waves source. This can be avoided by excluding lateral positions close to the excitation beamwidth to avoid diffraction effects within this region [40], [42]. The lateral excitation beamwidth can be approximated by *Fλ*. The diffraction effect is illustrated in Figure 6, where the wave arrival times versus lateral positions from experimental data measured on a homogeneous elastic phantom are shown. The first 3 lateral positions are within the excitation beamwidth, and the wave arrival times at these 3 positions are inconsistent with the linear trend between the arrival times and lateral positions measured beyond the shear wave near-field. Performing linear regression including the arrival times at the first 3 lateral positions will lead to negative SWS bias.

In addition, it is often necessary to use track locations further away from the ARFI excitation for SWS estimation in stiff materials. The fast traveling shear wave motion in stiff materials can be missed at track locations close to the ARFI excitation, due to PRI limitations and reverberation frames.

## VII. Other imaging scenarios

This manuscript has focused on the sequencing and postprocessing procedures of a MTL-SWEI sequence using a linear array L7-4 as an example. This section extends the analysis to curvilinear arrays and other types of ultrasound elasticity imaging (i.e., STL-SWEI and ARFI).

### A. Curvilinear arrays

When a curvilinear array is used for ARFI excitation and shear wave tracking, the shear wave can travel at oblique angles instead of propagating along the lateral position when the push beam does not originate from the center of the transducer. In this case, using TOF methods with the assumption of laterally traveling shear waves can result in bias in SWS estimation. Song *et al.* [43] described a 2D SWS estimation scheme using the C5-2v curvilinear array and the Verasonics system. This scheme measures the shear wave speed along both the lateral and axial dimensions (*V_x_* and *V_z_*), and reconstructs the true SWS of the shear wave traveling at the oblique angle.

In addition, for phantom imaging using curvilinear arrays, the coupling between the transducer and the phantom can lead to SWS errors. Due to the curved surface of curvilinear arrays, there is a gap between the outer elements of the array and the phantom surface when the transducer is in contact with the phantom. Water is commonly used as the coupling medium to fill the gap to avoid transmitting the sound directly into air. When the speed of sound of water is different from the speed of sound of the phantom, it can cause refraction at the water-phantom boundary and result in SWS bias [21]. Tissue-mimicking phantoms are usually made to have a speed of sound of 1540 m/s to match the average sound speed in tissue. The speed of sound in water is about 1490 m/s at room temperature [44]. To reduce the SWS bias caused by this speed of sound mismatch, it is recommended to use an alcohol (9.6% by volume) [45] or saline (4.5g NaCl in 100 ml of water) [46] solution to have a speed of sound at 1540 m/s for the coupling medium.

### B. STL-SWEI and ARFI sequences

For the MTL-SWEI sequence discussed so far, only 2 tx structures are used in the Verasonics programming script to define the transmit focal configurations. One tx structure is used to describe the plane wave tracking that insonifies a large FOV, the other one is used to describe the single focused push beam at the center of the transducer to perform the ARFI excitation. The repeated tracking pulses have identical transmit configurations in a MTL-SWEI sequence. In STL-SWEI and ARFI sequences, a larger number of tx structures is required in the programming script to describe the different push and track transmits. In STL sequences, multiple push beams are transmitted at different spatial locations, so that each push beam needs a unique tx structure to define the unique transmit aperture location. In ARFI sequences, multiple tx structures are required to define the track beams in addition to the push beams, as the track beams move along with the push beams spatially if focused track beams are used. Focused track beams instead of plane wave tracking are typically used to provide high tracking data quality in STL-SWEI and ARFI sequences, as the tracking beams in these sequences are not required to cover the same large FOV as in MTL-SWEI sequences.

Although this paper does not focus on STL-SWEI and ARFI sequences, we also provide example scripts for these two types of sequences for the C5-2 transducer. They can be found in the same Github repository that contains the MTL-SWEI sequence mentioned in Section II. setupc5_2shear_wave_stl.mis a STL-SWEI sequence, which has a single track beam located at the center below the transducer, and has 6 push beams evenly spaced between 2.5 °C and 10 °C. setupc5_2arfi.mis an ARFI sequence, which contains 16 push beams that evenly cover a field of view of 18.7 degrees.

The three example scripts we provide for the C5-2 transducer use different track transmit focal configurations. Diverging wave transmit was used for the MTL-SWEI sequence, so that each track transmit insonifies the entire lateral FOV to monitor the shear wave propagation. Diverging wave or plane wave transmit results in sub-optimal track data quality compared to focused tracking. Coherent plane wave compounding is typically used to improve the image quality of plane wave imaging at the expense of frame rate [47]. Focused track beams at 50 mm with an F/2 focal configurations were used in the STL-SWEI sequence, which requires a single track beam that monitors the tissue motion from multiple laterally-offset push beams. The use of focused track in STL sequences ensure high track data quality and low tissue displacement jitter. In addition, focused track beams with concurrent push and track foci are typically used in ARFI sequences, as ARFI imaging measures tissue displacement along the push axis. However, diverging wave tracking was used in the provided ARFI imaging sequence setupc5_2arfi.m, which provides both on-axis and off-axis tracking data. The user is able to obtain combined ARFI and MTL-SWEI data from this single ARFI sequence.

## VIII. Conclusions

This paper provides guidelines for setting up several ARFI and SWEI sequences for a linear and a curvilinear array using a Verasonics system, presents procedures of calibration and safety measurements, and demonstrates the data processing pipeline to calculate group SWS. The parameter selection of a SWEI sequence has been discussed in detail. Calibration of beam positions and scanner timing are important to avoid SWS bias. Transducer face heating measurement is recommended to avoid transducer damage, and acoustic and thermal output measurements are necessary before clinical studies. The data processing pipeline to calculate SWS involves 3 steps: tissue motion estimation, data filtering, and SWS estimation. The procedures of each step were discussed, with some simple guidelines to reduce bias in SWS estimation.

## Figures and Tables

**Table I:** List of symbols and abbreviations

Symbol	Name
*α*	Ultrasonic attenuation
*c*	Sound speed
*f*	Force density
*f_c_*	Track center frequency
ℱ	Fourier transform operation
*I*	Acoustic Intensity
*λ*	Wavelength
*ρ*	Density
*t*	Slow time
*u*	Particle displacement
*μ*	Shear modulus
*v*	Particle velocity
*ω*	Angular frequency
ARFI	Acoustic radiation force impulse
CUSE	Comb-push ultrasound shear elastography
DOF	Depth of field
FOV	Field of view
HPF	High pass filter
IQ	In-phase and quadrature data
LPF	Low pass filter
MTL	Multiple track location
NXcorr	Normalized cross-correlation
PRF	Pulse repetition frequency
PRI	Pulse repetition interval
RF	Radio frequency
RANSAC	Random sample consensus
ROC	Radius of curvature
SMURF	Spatially modulated ultrasonic radiation force
SNR	Signal-to-noise ratio
SSI	Supersonic shear imaging
STL	single track location
SWEI	Shear wave elasticity imaging
SWS	Shear wave speed
TOF	Time of flight

**Table II:** SWEI acquisition parameters and locations in MATLAB structures

Parameter	MATLAB struct	Default L7-4 values	C5-2 values
Push frequency	tw	5.2 MHz	2.4 MHz
Track frequency	tw	5.2 MHz	3.1 MHz
Push duration	tw	1000 cycles, 192 *μ*s	800 cycles, 333 *μ*s
Push focal configurations	tx	F/1.5 at 14.8 mm	F/2 at 50 mm
Track focal configurations	tx	Plane wave, full aperture	Diverging wave, full aperture
Receive configurations	receive		
Channel data sampling frequency	receive trans	20.8 MHz (5.2 × 4)	12.4 MHz (3.1 × 4)
Beamformed IQ data sampling frequency	pdata	0.25*λ*	0.25*λ*
Pulse repetition interval (PRI)	seqcontrol	100 *μ*s	200 *μ*s
Excitation voltages	Imaging GUI		
